# Astragaloside IV-PESV inhibits prostate cancer tumor growth by restoring gut microbiota and microbial metabolic homeostasis via the AGE-RAGE pathway

**DOI:** 10.1186/s12885-024-12167-z

**Published:** 2024-04-15

**Authors:** Xujun You, Junfeng Qiu, Qixin Li, Qing Zhang, Wen Sheng, Yiguo Cao, Wei Fu

**Affiliations:** 1 Department of Andrology, Shenzhen Bao’an Traditional Chinese Medicine Hospital, Guangzhou University of Chinese Medicine, 518101 Shenzhen, China; 2 Department of Andrology, Shenzhen Traditional Chinese Medicine Hospital, Guangzhou University of Chinese Medicine, 518033 Shenzhen, China; 3 School of Rehabilitation Medicine and Health Care, Hunan University of Medicine, 418000 Huaihua, China; 4 School of Traditional Chinese Medicine, Hunan University of Medicine, 418000 Huaihua, China; 5 Department of Urology Surgery, Shenzhen Bao’an Traditional Chinese Medicine Hospital, Guangzhou University of Chinese Medicine, 518101 Shenzhen, China

**Keywords:** Prostate cancer, Astragaloside IV, PESV, AGE-RAGE

## Abstract

**Background:**

Prostate cancer (PCa) is becoming the most common malignancy in men worldwide. We investigated the effect of astragaloside IV combined with PESV on the gut microbiota and metabolite of PCa mice and the process of treating PCa.

**Methods:**

Nude mice were genetically modified to develop tumors characteristic of PCa. The treatment of PCa mice involved the administration of a combination of astragaloside IV and peptides derived from scorpion venom (PESV). Feces were collected for both 16 S rDNA and metabolic analysis. Fecal supernatant was extracted and used for fecal transplantation in PCa mice. Tumor development was observed in both PCa mice and nude mice. Tumor histopathology was examined, and the expression of inflammatory factors and the AGE-RAGE axis in PCa tissues were analyzed.

**Results:**

PCa mice treated with Astragaloside IV in combination with PESV showed a significant reduction in tumor volume and weight, and stabilization of gut microbiota and metabolites. At the Genus level, significant differences were observed in *Porphyromonas*, *Corynebacterium*, *Arthromitus* and *Blautia*, and the differential metabolites were *PA16_016_0*, *Astragaloside+*, *Vitamin A acid*, *Nardosinone*, *a-Nortestoster*, *D-Pantethine*, *Hypoxanthine*, *Pregnenolone*, *cinnamic acid*, *Pyridoxa*, *Cirtruline* and *Xanthurenate*. There was a correlation between gut microbiota and metabolites. After the fecal transplantation, tumor growth was effectively suppressed in the PCa mice. Notably, both the mRNA and protein levels of the receptor for advanced glycation end products (RAGE) were significantly decreased. Furthermore, the expression of inflammatory factors, namely NF-κB, TNF-α, and IL-6, in the tumor tissues was significantly attenuated. Conversely, upregulation of RAGE led to increased inflammation and reversed tumor growth in the mice.

**Conclusion:**

Astragaloside IV combined with PESV could treat PCa by intervening in gut microbiota composition and metabolite by targeting RAGE.

**Supplementary Information:**

The online version contains supplementary material available at 10.1186/s12885-024-12167-z.

## Introduction

Prostate cancer (PCa) is one of the most common malignancies in men with high morbidity and mortality worldwide [1]. In 2020, there were more than 1,414,000 new cases of PCa worldwide [2]. PCa causes death, mainly due to incurable metastatic disease [3]. Many therapeutic strategies have offered the opportunity for the cure of PCa, including radical prostatectomy or radiation therapy [4]. Unfortunately, most PCa patients are often diagnosed in an advanced stage in which radical prostatectomy cannot be performed leading to poor prognosis. The 5-year relative survival rate for localized PCa is close to 100%, compared to 30% for advanced metastatic prostate cancer (mPCa) [5]. Therefore, the search for new treatment options continues.

Traditional Chinese medicine (TCM) is gaining increasing recognition worldwide for its low toxicity, low side effects and good tolerability [6]. It is worth noting that there is evidence that TCM plays an indispensable role in the prevention and treatment of cancer, preventing the occurrence of tumors, reducing the toxic and side effects of chemotherapy and other treatments, and reducing the recurrence and metastasis of tumors, which can be reduced [7]. Astragaloside IV (3-O-β-D-Xylopyranosyl-6-O-β-D-glucopyranosylcycloastragenol) is a natural saponin extracted from Astragalus membranaceus with anti-inflammatory, anticancer, antioxidant and immunomodulatory properties [8]. Recent studies have shown that scorpion venom, the main active ingredient in scorpions, has anti-cancer properties. For example, the venom of the scorpion Androctonus amoreux has significant cytotoxic and antiproliferative effects on PCa cells [9]. Scorpion venom peptide (PESV) inhibits the proliferation of PCa cells [10]. However, the mechanism of action of astragaloside IV drugs for the treatment of PCa is unknown.

Astragaloside IV also effectively reversed intestinal autophagy and oxidative stress induced by the gut microbiota in mice during the onset of acute ischemic stroke [11]. It has been demonstrated that the distribution of astragaloside IV in the enterohepatic circulation and its therapeutic effects are regulated by the gut microbiota [12]. PCa is clinically associated with dietary factors and nutrients, such as dairy products and fats [13]. The composition of the gut microbiota is highly influenced by dietary habits and body size, and is involved in host inflammatory and immune responses [14]. Animal studies have shown that a high-fat diet and obesity can promote local prostate inflammation and proliferation of PCa cells [15]. Furthermore, it has been reported that the gut microbiota of atherosclerotic mice is disrupted by medication, resulting in significant inhibition of the AGE-receptor (RAGE) pathway and reduction in atheromatous plaques [16]. In our preliminary experiments, the pathways differentially enriched to the gut microbiota were the AGE-RAGE pathway [17]. These results suggest that diet and other extrinsic factors alter gut microbiota and may be involved in PCa progression through multiple mechanisms and affect gut microbiota diversity through the AGE-RAGE pathway. These results suggested that diet and other exogenous factors can alter the gut microbiota and may be involved in PCa progression through multiple mechanisms. This suggested that in PCa, Astragaloside IV can interfere with gut microbiota diversity through the AGE-RAGE pathway.

AGE is non-enzymatic protein modifications that arise during normal aging [18]. RAGE is overexpressed in several tumor types, including PCa [19]. It has been suggested that interactions between AGE and RAGE are implicated in the development, growth, and metastasis of various types of tumors, such as PCa [20]. Deficiency in RAGE leads to reduced levels of pro-inflammatory cytokines IL-6, TNF-α, and NF-κB in the bloodstream [21]. However, the molecular mechanisms regulating these effects associated with AGE-RAGE in PCa cells remain unclear.

Although the results of previous studies supported that gut microbiota has an effect on the AGE-RAGE pathway, it has not been demonstrated whether astragaloside IV-PESV can regulate the AGE-RAGE pathway to inhibit PCa tumor growth by improving gut microbiota and metabolic disorders. The aim of this study was to demonstrate the combination of astragaloside IV-PESV for the treatment of PCa through in vivo experiments, providing a basis for the treatment of prostate cancer. Our research on gut microbiota and PCa is presented as a Graphical abstract (Figure S1).

## Materials and methods

### Experimental material

16 ± 2 g BALB/c 100 nude mice were purchased from Hunan Slack Jingda Experimental Animal Co., Ltd. The experiments on animals were approved by the Experimental Animal Ethics Committee of Guangzhou University of Chinese Medicine (NO. 20210224026). Animals were handled in accordance with the “Specifications for the Use of Experimental Animals” promulgated by the Ministry of Science and Technology in 2006. The PCa cell system PC-3(AW-CCH111, Changsha, China) was purchased from Abiowell Co., LTD. All plasmids were purchased from Honorgene Co., LTD.

### Tumor formation in nude mice

Male BALB/c nude mice of 6 weeks old size were divided into 10 groups of 10 mice each. PC-3 2 × 10^8^ units/mL (0.1 mL/each) of 2 × 10^7^ was injected into the left axilla of each mouse for a 4-week experimental period. Mice were grouped, and dosing was started one week after implantation: astragaloside was administered at a dose of 40 mg/kg by gavage. Scorpion venom polypeptide was administered at a dose of 1.2 mg/kg by intraperitoneal injection [17]. The solvent was saline, the Control group was given the same volume of distilled water by gavage, and the change in tumor volume was detected every 4 days. Four weeks later, the animals were treated, and the tumor tissues were removed and weighed. Feces from astragaloside IV-PESV-treated nude mice were collected, snap-frozen and stored at -80 °C. PC-3 cell suspensions with empty vector (OverExp-vector) or overexpression RAGE (oe-RAGE) transfected plasmids were prepared and 2 × 10^8^ units/mL (0.1 mL/each) were injected into the left axilla of each mouse for a 4-week experimental period, and mice were given an antibiotic cocktail by gavage one week after implantation containing ampicillin (0.1 mg/mL), streptomycin (0.5 mg/mL), and colistin (0.1 mg/mL) to ablate gut microbiota by gavage for 4 days and normal drinking water for 2 days. The Fecal microbiota transplantation (FMT)-astragaloside IV-PESV group, FMT-astragaloside IV-PESV + oe-NC group, and FMT-astragaloside IV-PESV + oe-RAGE group were given 0.1 mL of fecal supernatant by gavage every day for 14 days. The Control group was given 0.1 mL of sterile saline by gavage every day for 14 days. Tumor volume changes were measured every 4 days. At the end of the experiment, the mice were euthanized with an overdose of pentobarbital (250 mg/kg intraperitoneal injection). After confirming that the mice had died due to respiratory arrest and cardiac arrest, the tumor tissue was removed and weighed.

### Hematoxylin-eosin(HE) staining

Hematoxylin (AWI0001a, Abiowell, China) was stained for 1 ∼ 10 min, rinsed with distilled water and returned to blue in PBS (AWI0129a, Abiowell, China). Eosin (AWI0029a, Abiowell, China) was stained for 1 ∼ 5 min and rinsed with distilled water. Gradient alcohol (95–100%) was used for dehydration for 5 min per stage. Neutral gum (AWI0238a, Abiowell, China) was sealed and observed by microscopy (BA210T, Motic, China).

### Immunocytochemistry (IHC)

The slices were baked at 60 °C for 12 h. For thermal repair of antigens: the sections were immersed in 0.01 M citrate buffer (AWI0206a, Abiowell, China) (pH 6.0), heated in a microwave oven to boiling and then disconnected, cooked continuously for 20 min, then cooled for 20 min After the microwave oven was heated to boiling and then disconnected, the microwave oven was heated to boiling and then disconnected. After cooling, wash the sample three times with 0.01 M PBS (pH 7.2 ∼ 7.6) for 3 min each time. Next, add the primary antibody Ki67 (ab16667, Abcam, UK) dropwise and let it incubate overnight at 4 °C. 50–100 µL of anti-Rabbit-IgG antibody-HRP multimers were added by incubating at 37 °C for 30 min. Chromogenic DAB working solution (ZLI-9018, ZSbio, Beijing) 50 ∼ 100 µL was added dropwise. Hematoxylin (AWI0001a, Abiowell, China) was re-stained for 5 ∼ 10 min, rinsed with distilled water, and returned to blue in PBS. Each stage was dehydrated in alcohol (60–100%) for 5 min each. Slices were observed by microscopically.

### PPI network construction

After collecting 6 groups of mice fecal samples, 6 samples from each group will be collected. Library construction and library testing will be performed, and the libraries that pass the testing will be sequenced using Illumina PE150. The downstream data obtained from sequencing will be used for later information analysis. Species notes will be made for each OTU representative sequence. The Raw Data obtained from sequencing will be used for post-information analysis.

### UPLC-MS

The extracted feces were placed in a 1.5 mL centrifuge tube. Liquid nitrogen was added to the centrifuge tube and, using a plastic hammer, the excretion was rapidly mashed. The extract and the powdered intestinal excretion were left on ice for 10–15 min to allow sufficient reaction. After centrifugation at high speed and low temperature (16,000 g, 4 ºC) for 10 min, the resulting supernatant contained small molecule metabolites. The supernatant was then transferred to a new centrifuge tube and dried using nitrogen. Following the completion of each run, the injection needle was cleaned for 5 s. The UHPLC system (1290, Agilent Technologies) combined with TripleTOF 6600 (Q-TOF, AB Sciex) and QTOF 6550 were analyzed on Waters HSS T3 column (100 × 2.1 mm, 1.7 μm).

### Quantitative real-time PCR(qRT-PCR)

Total The RNA samples were thawed on ice and diluted according to the kit instructions. The RNA samples were added to the reverse transcription reaction system according to the instructions of the reverse transcription kit, and the cDNA template was reverse transcribed. The cDNA was mixed with the PCR kit and PCR amplification was performed according to the conditions set by the PCR program. The internal reference of primer was β-actin, and the primer sequence was shown in Table 1. Relative quantitative method (2^−ΔΔCt^ method) was used to calculate the relative transcription levels of target genes: ΔΔCt=ΔCt experimental group - ΔCt control group, ΔCt = Ct (target gene) -Ct (β-actin).

**Table 1:** PCR primer sequence

Gene	Sequences (5’-3’)
RAGE	F: GCTCAAAACATCACAGCCCG
	R: ACCTTCCAAGCTTCTGTCCG
β-actin	F: ACCCTGAAGTACCCCATCGAG
	R: AGCACAGCCTGGATAGCAAC

### Western blot

The sample to be assayed is added to protein extraction buffer and centrifuged to remove cellular debris. Depending on the required number of gel wells and the size of the protein being tested, an SDS-PAGE gel is prepared and heated until melted. Electrophoresis is then conducted to separate the proteins on the gel. For primary antibodies, we used rabbit anti-RAGE (1:1000, ab216329, Abcam), rabbit anti-NF-κ (1:5000, ab32536, Abcam), rabbit anti-TNF-α (1:1000, 17590-1-AP, Proteintech) and rabbit anti-IL-6 (1:1000, ab259341, Abcam). TBST were rinsed 3 times for 10 min each. HRP goat anti-mouse IgG (1:5,0000, SA00001-1, Proteintech) was then incubated.

### Statistical analysis

All measurement data are expressed as mean ± standard deviation (SD). Each test was repeated independently three times. All data were analyzed by using SPSS 26.0 software (IBM, Armonk, NY, USA). The unpaired student’s t-test was used to compare the data of two groups that were not a one-to-one correspondence. One-way ANOVA and Tukey’s post-hoc test were used to compare data among three groups. The difference was statistically significant at *P* < 0.05.

## Results

### Astragaloside IV-PESV can reshape gut microbiota homeostasis in PCa

**Fig. 1:**
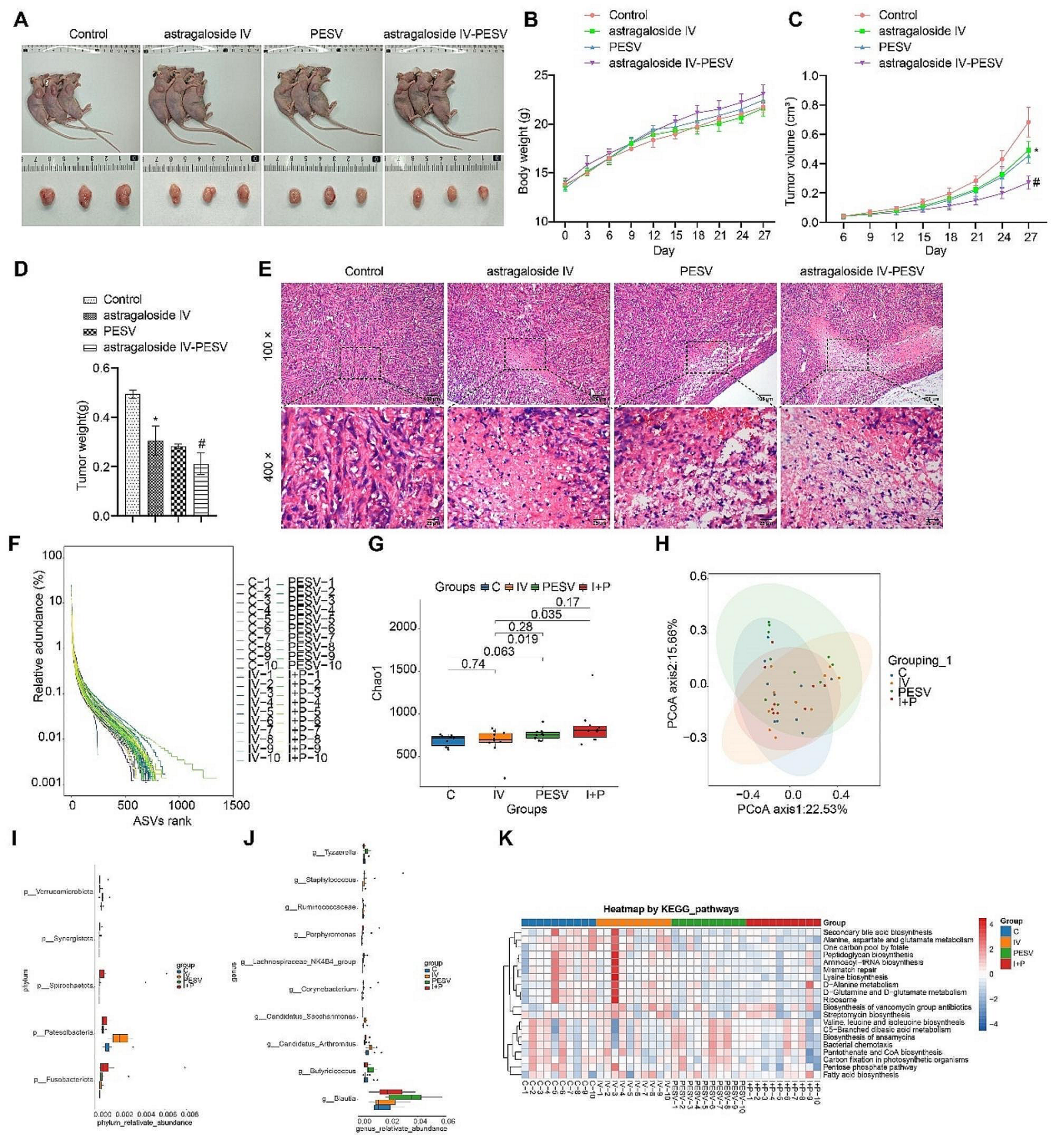
Aastragaloside IV-PESV can affect PCa gut microbiota. **A**. Images of each group of nude mice and their tumors. **B**. Body weight. **C**. Plots of tumor volume change over four weeks. **D**. Tumor mass of each group. **E**. HE staining of tumor tissue of each group. **F**. Rand-Abandance curves showed the species richness of each group. **G**. Differences between species abundance of each group. **H**. Differences in the composition of groups of colonies by principal component analysis. **I**. Groups of colonies that differed more at the Phylum level. **J**. Bacteria that differed more among groups at the Genus level. **K**. KEGG was to analyse of pathway correlation heat map for four groups of enrichment. *, *P* < 0.05 compared with the Control group. #, *P* < 0.05 compared with the PESV group

We constructed PCa nude mice into tumors and used astragaloside and scorpion venom peptide to intervene in the nude mice. The tumor volume changes were detected every 3 days during this period, and the nude mice were sacrificed after 4 weeks and the tumors were removed and weighed. Compared with the Control group, the tumor volume mass decreased in the astragaloside IV, PESV and astragaloside IV-PESV groups and was lowest in the astragaloside IV + PESV group. There was no significant change in body weight of the mice (Fig. 1A–D). From the HE staining results, the tumor tissues of nude mice subjected to astagaloside IV-PESV intervention showed increased vacuolation and damage to PCa cells. It implied that astragaloside IV-PESV can effectively inhibit the development of PCa (Fig. 1E). To investigate whether astragaloside IV-PESV could affect the intestinal metabolism of PCa mice, we collected feces from nude mice for intestinal microbial and metabolic analysis. A Rank-Abundance curve showed that the Control group was the most sparse and the astragaloside IV-PESV group was the most abundant (Fig. 1F). The difference in abundance was most significant in the astagaloside IV-PESV group compared to the Control group. The combined intervention was more significant than the two groups with a single intervention (Fig. 1G). The results of the principal component analysis showed some similarities and differences among the four groups. The first principal component accounted for 22.53%, and the second principal component accounted for 15.66% (Fig. 1H). At the Phylum level, the groups with significant differences among the four groups were *Verrucomicrobiota, Synergistota, Patescibacteria, Fusobacteriota* and so on. At the Genus level, the groups with significant differences among the four groups were *Porphyromonas, Corynebacterium, Arthromitus, Blautia* and so on (Fig. 1I and J). All gut microbiota we present again in the form of a list (Table S1). Finally, we analyzed the pathway correlations between the four groups. KEGG heat map showed that the enriched pathways had different correlations between the four groups (Fig. 1K). In conclusion, the astragaloside IV + PESV group had a greater effect on PCa tumor development and gut microbiota in nude mice.

### Astragaloside IV-PESV can restore metabolic homeostasis in PCa

**Fig. 2:**
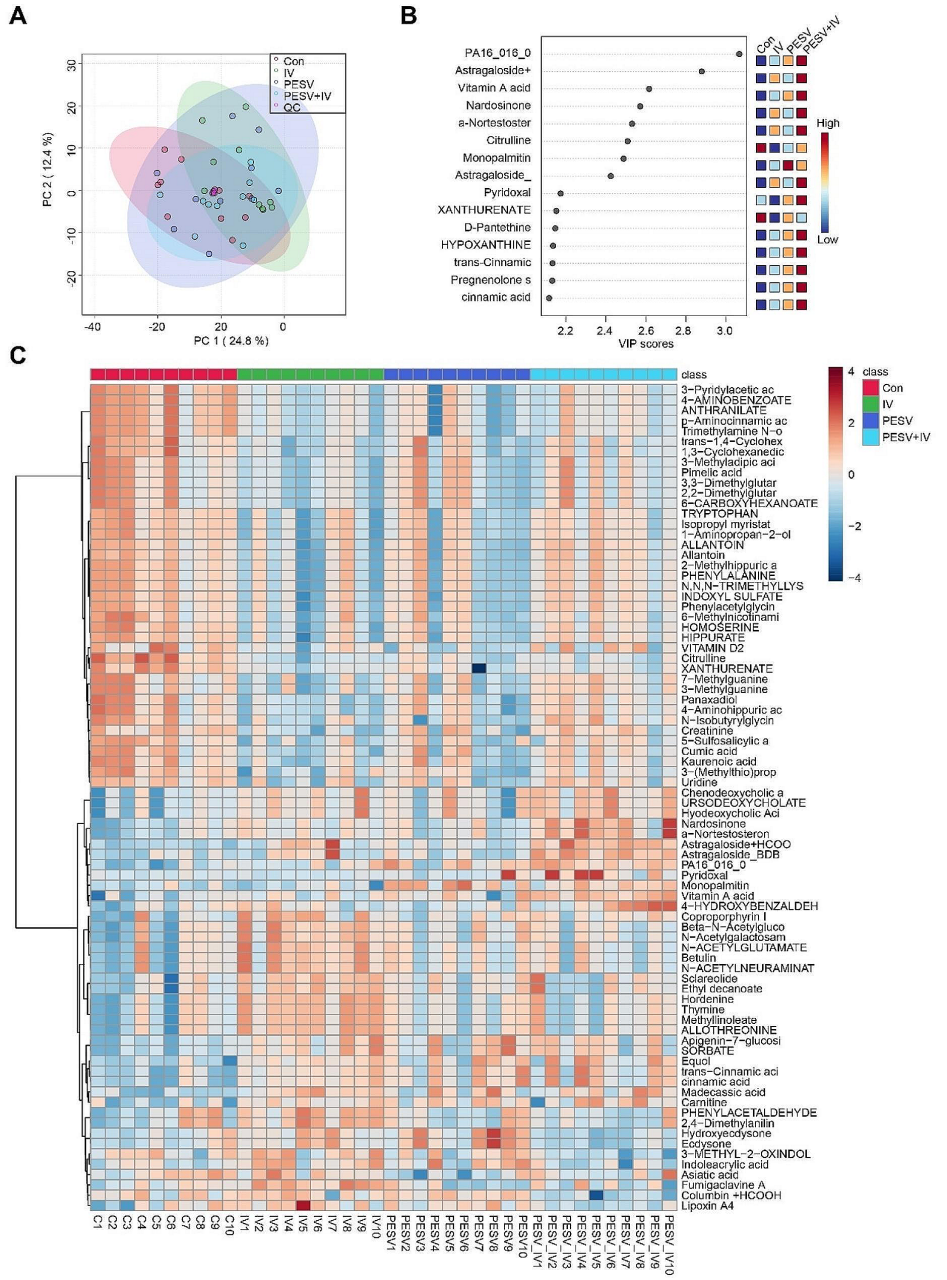
Astragaloside IV-PESV can affect the metabolic homeostasis of PCa. **A**. Principal component analysis of the differences in the metabolites of the four groups. **B**. Bubble diagram was used to show the 15 differential metabolites. **C**. Heat map was used to show the Top80 metabolites with differences in the four groups

After the above results showed that Astragaloside IV-PESV could affect PCa gut microbial diversity, we then examined the types of metabolites in the feces. The results of the principal component analysis showed that there were common components among the four groups, and a number of differences existed. The first main component accounted for 24.8% and the second main component accounted for 12.4. The data implied that there were some differences in metabolites between the four groups (Fig. 2A). As seen according to VIP scores, in the PESV + IV group, *metabolites PA16_016_0, Astragaloside+, Vitamin A acid, Nardosinone, a-Nortestoster, D-Pantethine, Hypoxanthine, Pregnenolone, cinnamic acid, Pyridoxal* were significantly elevated, while *Cirtruline* and *Xanthurenate* were significantly decreased (Fig. 2B). Heat maps were produced based on the four groups of differential metabolites Top80. As shown in the figure, the differences between the four groups were significant (Fig. 2C). This showed that Astragaloside IV-PESV has a significant effect on intestinal microbial metabolism.

### Correlation analysis of gut microbiota and metabolite

**Fig. 3:**
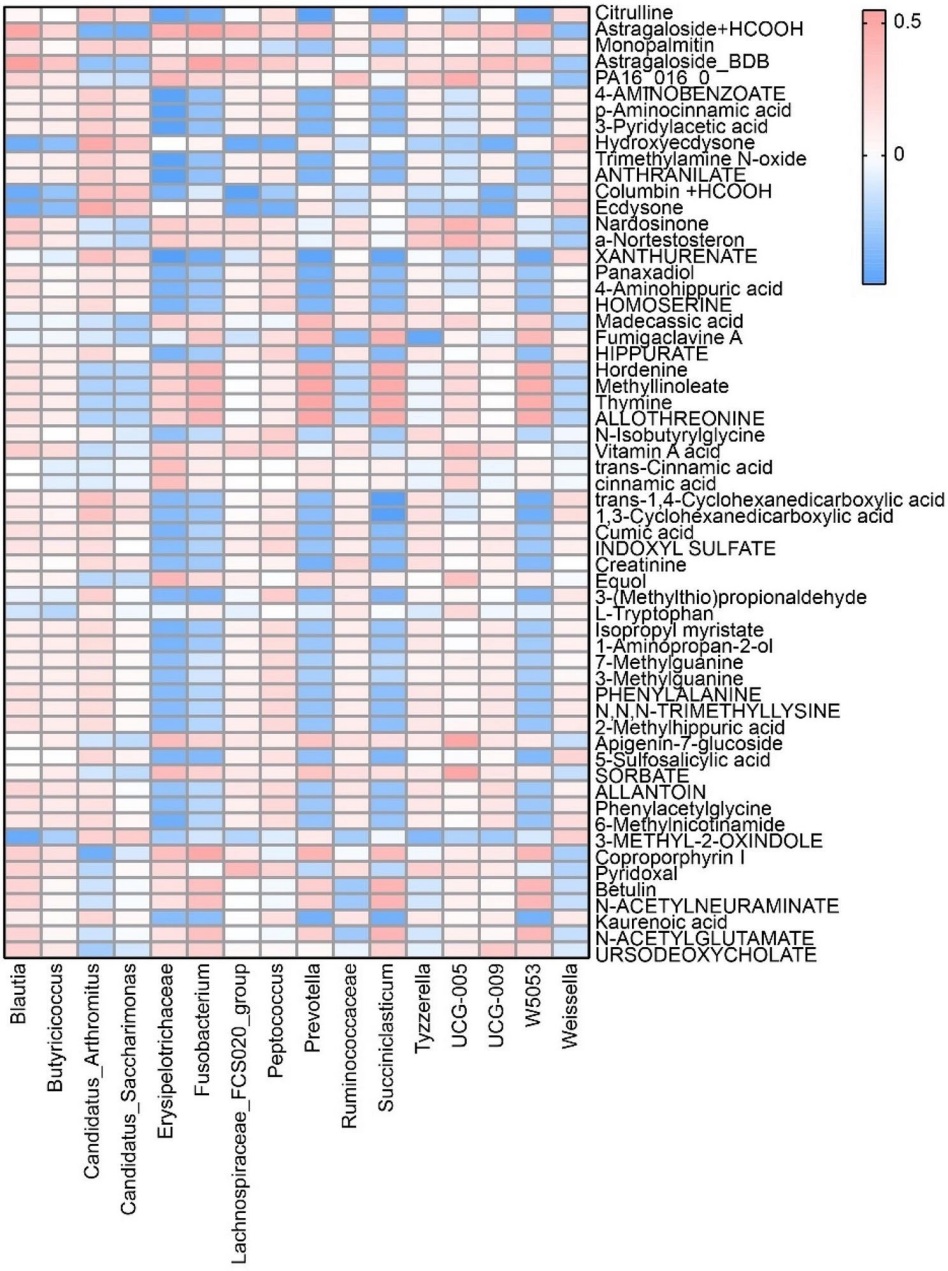
Heat map displayed the correlation between 16 gut microorganisms and 60 differential metabolites

In order to explore the correlation between intestinal microbes and metabolites, we analyzed the correlation between top 60 metabolites and top 16 intestinal microbes. *Erysipelotrichaceae, Fusobacterium, Prevotella, Succiniclasticum* and *W5053* were negatively correlated with about 32 metabolites. *Blautia, Butyricicoccus, Arthromitus, Saccharimonas, FCS020, Peptococcus, Ruminococcaceae, Tyzzerella, UCG-005, UCG-009* and *Weissella* were positively correlated with 28 different metabolites (Fig. 3). These results indicated that gut microbiota had an effect on the level of intestinal metabolites.

### Astragaloside IV-PESV can affect the AGE-RAGE pathway

**Fig. 4:**
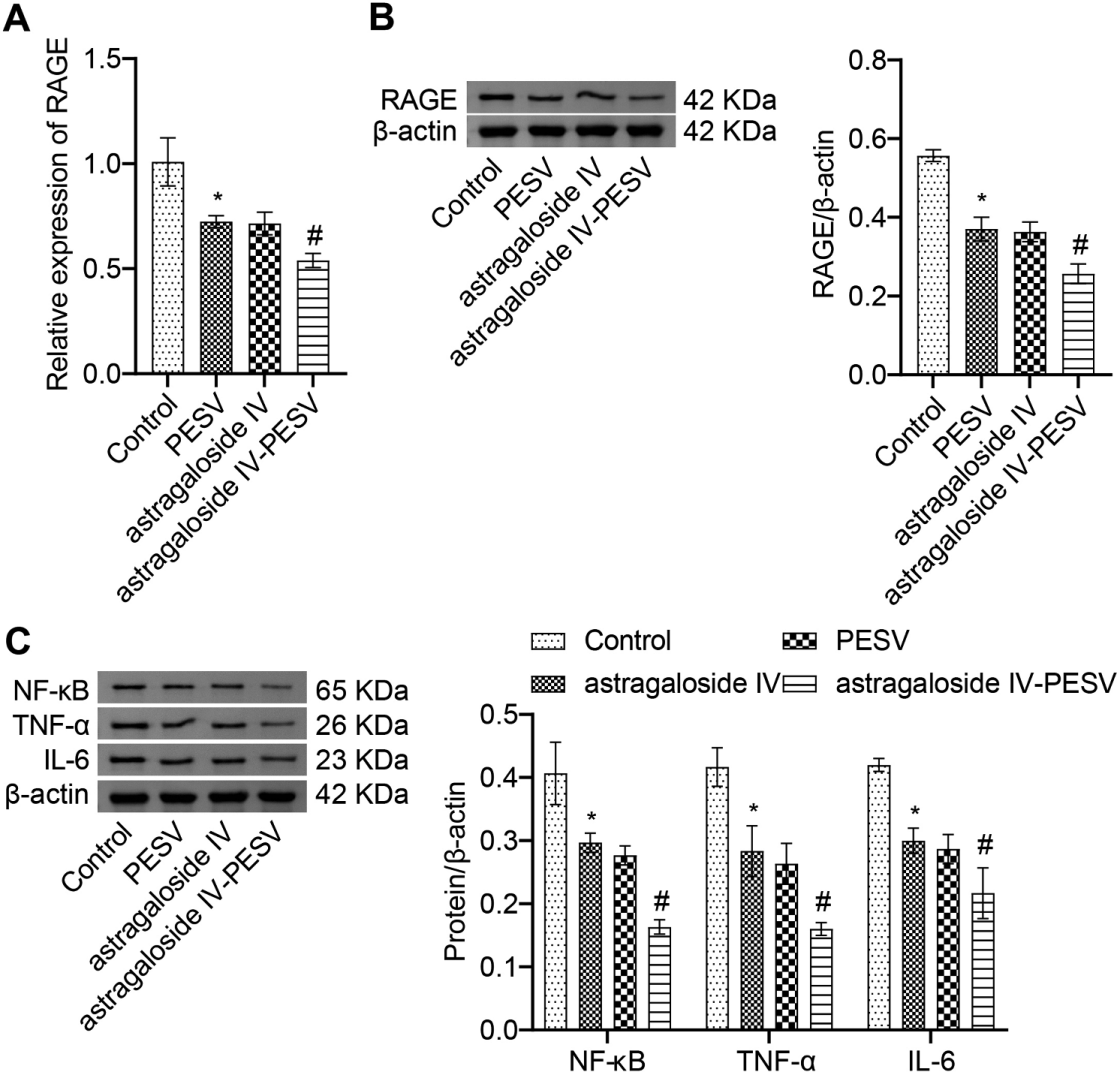
Astragaloside IV-PESV can inhibit the expression of RAGE. **A**. qRT-PCR was used to detect the expression of mRNA of RAGE in each group. **B**. Western blot was used to detect the protein expression of RAGE in each group of PCa tissues. **C**. Western blot was used to detect the protein expression of inflammatory factors NF-κB, TNF-α and IL-6 in each group. *, *P* < 0.05 compared with the Control group. #, *P* < 0.05 compared with the PESV group

In previous experiments, we enriched that the AGE-RAGE pathway might be associated with PCa progression [17], but no experiments were performed. Therefore, in this paper we collected PCa tissue samples for AGE-RAGE validation. Compared with the Control group, the mRNA and protein expression of RAGE were decreased in the PESV group, astragaloside IV group, and astragaloside IV-PESV group. The mRNA and protein expressions of RAGE were most significantly decreased in the astragaloside IV-PESV group (Fig. 4A and B). The inflammatory factors NF-κB, TNF-α and IL-6 were also decreased by astragaloside IV and PESV treatment. The expression of NF-κB, TNF-α and IL-6 was lowest in PCa tissues co-treated with astragaloside IV and PESV (Fig. 4C). In short, astragaloside IV-PESV inhibited AGE-RAGE pathway and inflammatory response.

### Astragaloside IV-PESV inhibits tumor growth through the gut microbiota

**Fig. 5:**
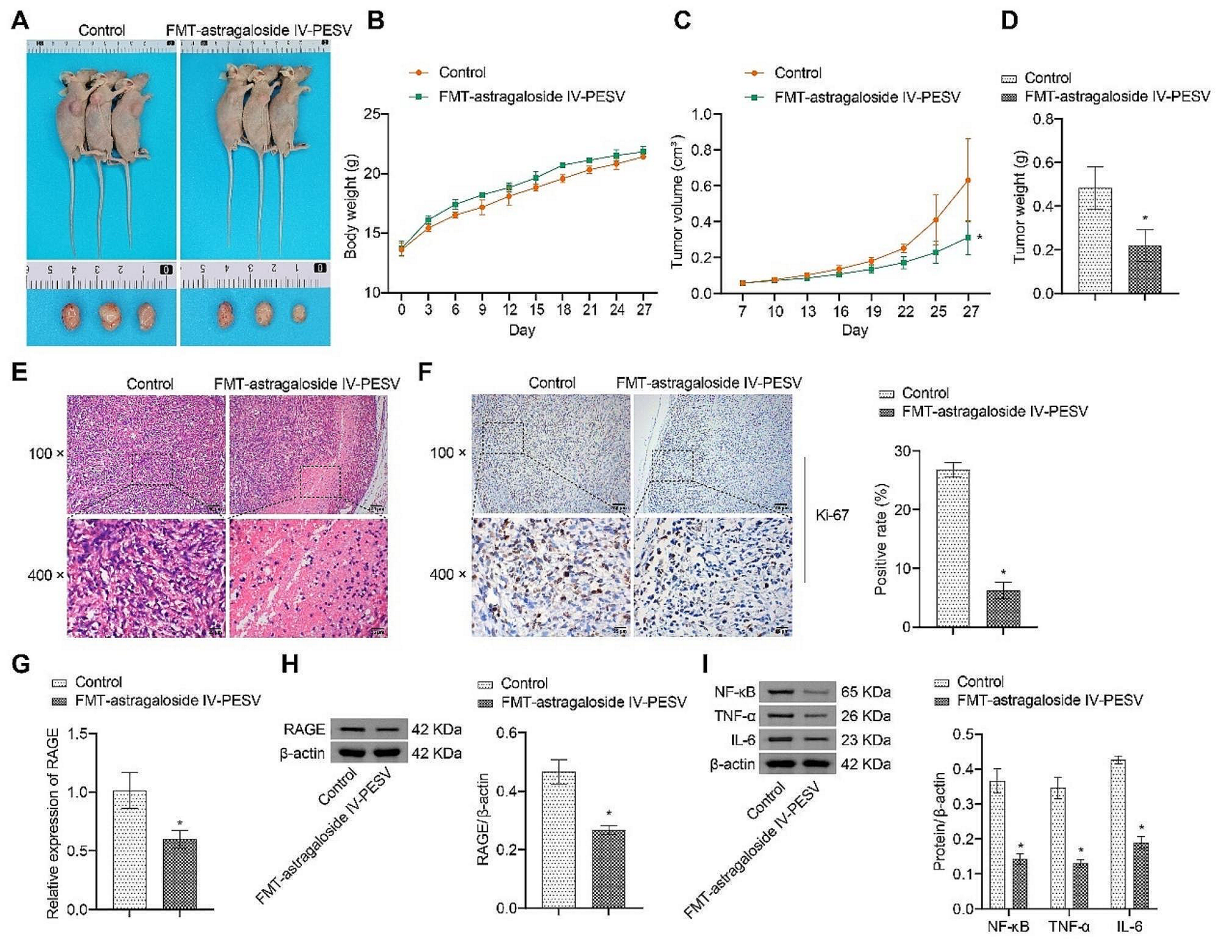
Astragaloside IV-PESV could affect the composition of the gut microbiota of PCa mice. **A**. PCa nude mice and tumor plots. **B**. Body weight. **C**. The data of four-week tumor volume. **D**. Tumor mass. **E**. HE staining plots of tumor tissues. **F**. IHC was used to analyze the Ki67 positive rate of tumor tissues. **G**. The mRNA level of RAGE in tumor tissues. **H**.The protein level of RAGE in tumor tissues. **I**. The protein level of NF-κB, TNF-α and IL-6 in tumor tissues. Western blot was used to detect the protein expression levels of NF-κB, TNF-α and IL-6 in tumor tissues. *, *P* < 0.05 compared with the Control group

The above experimental results indicated that Astragaloside IV-PESV was effective on PCa gut microbiota homeostasis, so we took fecal transplantation to investigate whether Astragaloside IV-PESV could inhibit PCa tumor growth through gut microbiota. In terms of tumor volume and mass, the tumor volume and mass of PCa nude mice transplanted with Astragaloside IV-PESV feces were significantly reduced. There was no significant change in body weight of the mice (Fig. 5A–D). Compared with the Control group, vacuoles appeared in the tumor tissue and tumor cells were sparse in the FMT-astragaloside IV-PESV (Fig. 5E). Ki67 positivity was decreased (Fig. 5F). This implies that PCa tumor growth was inhibited. RAGE and inflammatory factors NF-κB, TNF-α and IL-6 were significantly decreased in the FMT-astragaloside IV-PESV group compared to the Control group (Fig. 5G–I). In conclusion, Astragaloside IV-PESV can inhibit PCa tumor growth by affecting AGE-RAGE pathway through gut microbiota.

### Astragaloside IV-PESV can inhibit tumor growth in a gut microbiota-dependent manner via the AGE-RAGE pathway

**Fig. 6:**
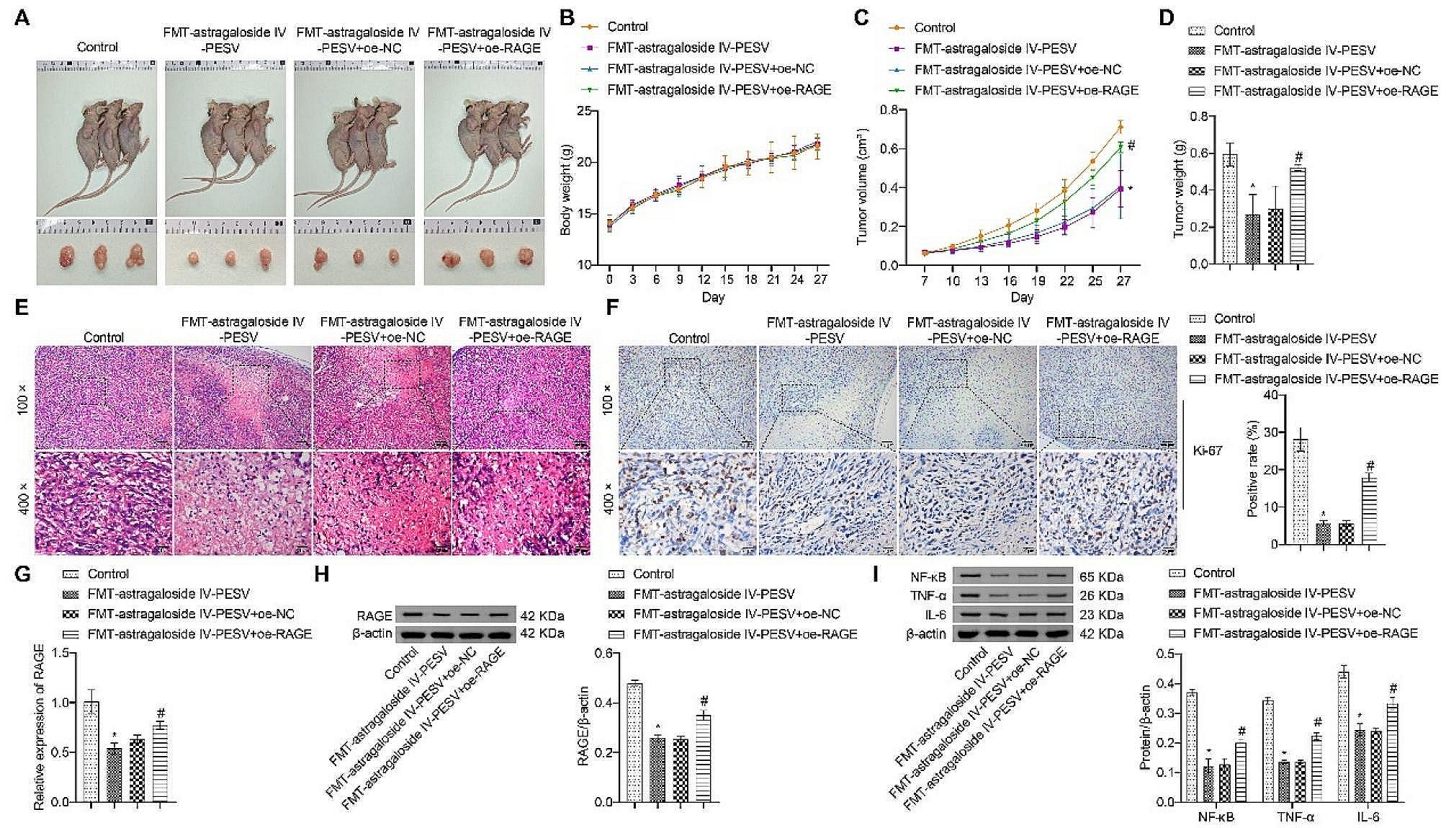
The AGE-RAGE pathway can promote the development of PCa. **A**. The images of nude mice and tumors. **B**. Line graph of tumor volume. **C**. Histogram of tumor mass. **D**. HE staining of tumor tissues. **E**. Positive rate of Ki67. **F**. mRNA levels of RAGE in tumor tissues. **G**. Protein levels of RAGE in tumor tissues. **H**. Protein expression levels of NF-κB, TNF-α and IL-6 in tumor tissues. *, *P* < 0.05 compared with the Control group. #, *P* < 0.05 compared with the FMT-astragaloside IV-PESV + oe-NC group

The above results suggested that Astragaloside IV-PESV fecal transplantation is effective in reducing tumor growth rate. This process may be related to the AGE-RAGE pathway. To test this hypothesis, we verified whether the AGE-RAGE pathway could affect the progression of PCa by rescue experiments. Compared with the FMT-astragaloside IV-PESV + oeNC group, the tumor growth was accelerated and of higher quality in the FMT-astragaloside IV-PESV + oe-RAGE group (Fig. 6A-C). Vacuoles were reduced in PCa tumor tissues (Fig. 6D), and the Ki67 positivity rate was increased (Fig. 6E). This suggested that PCa tumor growth was accelerated after overexpression of RAGE. qRT-PCR and Western blot data showed that compared with the FMT-astragaloside IV-PESV + oe-NC group, RAGE and inflammatory factors NF-κB, TNF-α, IL-6 were significantly higher in the FMT-astragaloside IV-PESV + oe-RAGE group (Fig. 6F–H). In conclusion, activation of AGE-RAGE pathway can promote the development of PCa.

## Discussion

In this study, we investigated the progression of PCa in mice by PESV, the main bioactive component of astragaloside and scorpion venom. These data supported the hypothesis that astragaloside IV and PESV may be effective chemopreventive/intervention agents for prostate cancer. Our results clearly demonstrated that astragaloside IV and PESV stabilize gut microbiota diversity and metabolism, inhibit tumor growth, and promote cancer cell death in PCa mice. This process is achieved by regulating the AGE-RAGE pathway.

Plant and animal herbs are rich sources of gene regulators for the prevention and treatment of cancer [17]. Herbs containing Astragalus have better anti-gastric cancer efficacy in chemotherapy patients [22]. In particular, Astragaloside IV, one of the main components of triterpenoid saponins, has been shown to be a key component of the anti-cancer effects of Astragalus in cancers such as breast, lung, and gastric cancers [23]. However, there is a lack of research on the role of astragaloside in PCa through regulation of autophagy. In recent years, Studies of scorpions have been focused on scorpion venom [24]. Scorpion venom contains complex bioactive peptides and PESV is extracted from scorpion venom. PESV has been shown to be a promising promising anti-cancer agent [25]. PESV mediates the inhibition of hepatocellular carcinoma through upregulation of NK cell activity [26]. PESV can regulate PI3K/AKT/mTOR pathway by inducing autophagy of hepatocellular carcinoma cells to play an antitumor role [27]. Our study was the first to confirm that astragaloside IV-PESV inhibited PCa tumor growth. 16s rDNA and metabolic results showed that astragaloside IV-PESV could interfere with gut microbiota and metabolites of PCa mice.

Abnormal alterations of gut microbiota are closely associated with various pathogenic diseases. Modulation of the structure and diversity of the gut microbiota is considered as a promising new approach for the prevention and treatment of diseases [28]. For example, *omega − 3 polyunsaturated fatty acids* have recently been reported to have an important regulatory role in the development of the gut microbiota, which may be associated with neurodevelopment in adolescence and adulthood [29]. The health benefits of many gut microbiota regulators, such as the major response genes MyD88 and taurine, have been recently investigated [30]. We noted a significant recovery of gut microbiota in the colon tissue of PCa mice, including *Saccharimonas, Arthromitus* and *Ruminococcaceae*, after administration of astragaloside IV-PESV. *ruminococcaceae* was associated with a reduced risk of liver cancer [31]. The results of this study demonstrate that astragaloside IV is a potent gut microbiota modulator, making the herb a reliable source for screening for effective therapeutic modulators of the gut microbiota. This also suggests that there is a possibility. In the present study, we detected a normalization of intestinal metabolites such as *3-Pyridylacetic, p-Aminocinnamic, 3-Methyladipic* and *Pimelic acid* in PCa mice after astragaloside IV treatment.

The prolonged presence of AGEs brings about a variety of damages leading to metabolic dysfunction and diseases involving inflammation and oxidative stress [32]. These strategies include multifunctional effects such as inhibition of AGEs by polyphenols, blockade of RAGE-ligand interactions, modulation of gut microbiota abundance and diversity and alleviation of gut inflammation to delay or prevent the development of neurodegenerative diseases [21]. It demonstrated that dietary AGEs could induce TNF-α secretion from human macrophage-like cells and activated macrophages to generate more AGEs [33, 34]. This suggested that AGEs could act as an accelerator to induce inflammatory response via macrophage activation. It showed that AGE–BSA significantly enhanced IL-6 production from monocytes/macrophages, but suppressed Th1 (IL-2 and IFN-γ) and Th2 (IL-10) gene expression [35]. Enhanced monocytic IL-6 production was proven via MAPK-ERK and MyD88- transduced NF-κB p50 signaling pathways [36]. Our results showed that astragaloside IV-PESV was able to inhibit tumor growth by inhibiting NF-κB, TNF-α and IL-6. Analysis of the results of tumor formation in nude mice confirmed that the effect was induced through the AGE-RAGE axis by the action of astragaloside IV-PESV. In the future, we plan to collect clinical samples for 16 S and metabolic analyses of PCa. It is worth further exploring whether the expression of the AGE-RAGE axis in the clinic is consistent with the results of the mouse experiments. Furthermore, when the experimental conditions permit, we will also conduct further research on the drug half-life at the clinical level.

## Conclusion

Astragaloside IV-PESV can improve the composition of gut microbiota and inhibit the expression of inflammatory factors in PCa rats. Astragaloside IV-PESV can regulate gut microbiota and reduce the growth of PCa tumors by inhibiting the AGE-RAGE axis. Astragaloside IV-PESV has potential value as a treatment for PCa-related diseases.

### Electronic supplementary material

Below is the link to the electronic supplementary material.

**Supplementary Figure S1:** Astragaloside IV combined with PESV could treat PCa by intervening in gut microbiota composition and metabolite by targeting AGE-RAGE

**Supplementary Table S1:** Gut microbiota at phylum level and genus level

Supplementary Material 3

## Data Availability

The datasets generated and/or analysed during the current study are available in the NCBI BioProject repository, [https://www.ncbi.nlm.nih.gov/bioproject/PRJNA1011017].
