# Supplementary figures and images for: Astragaloside IV-PESV inhibits prostate cancer tumor growth by restoring gut microbiota and microbial metabolic homeostasis via the AGE-RAGE pathway
Source: BMC Cancer. 2024 Apr 15;24:472. doi: 10.1186/s12885-024-12167-z (PMC11017490; doi:10.1186/s12885-024-12167-z)

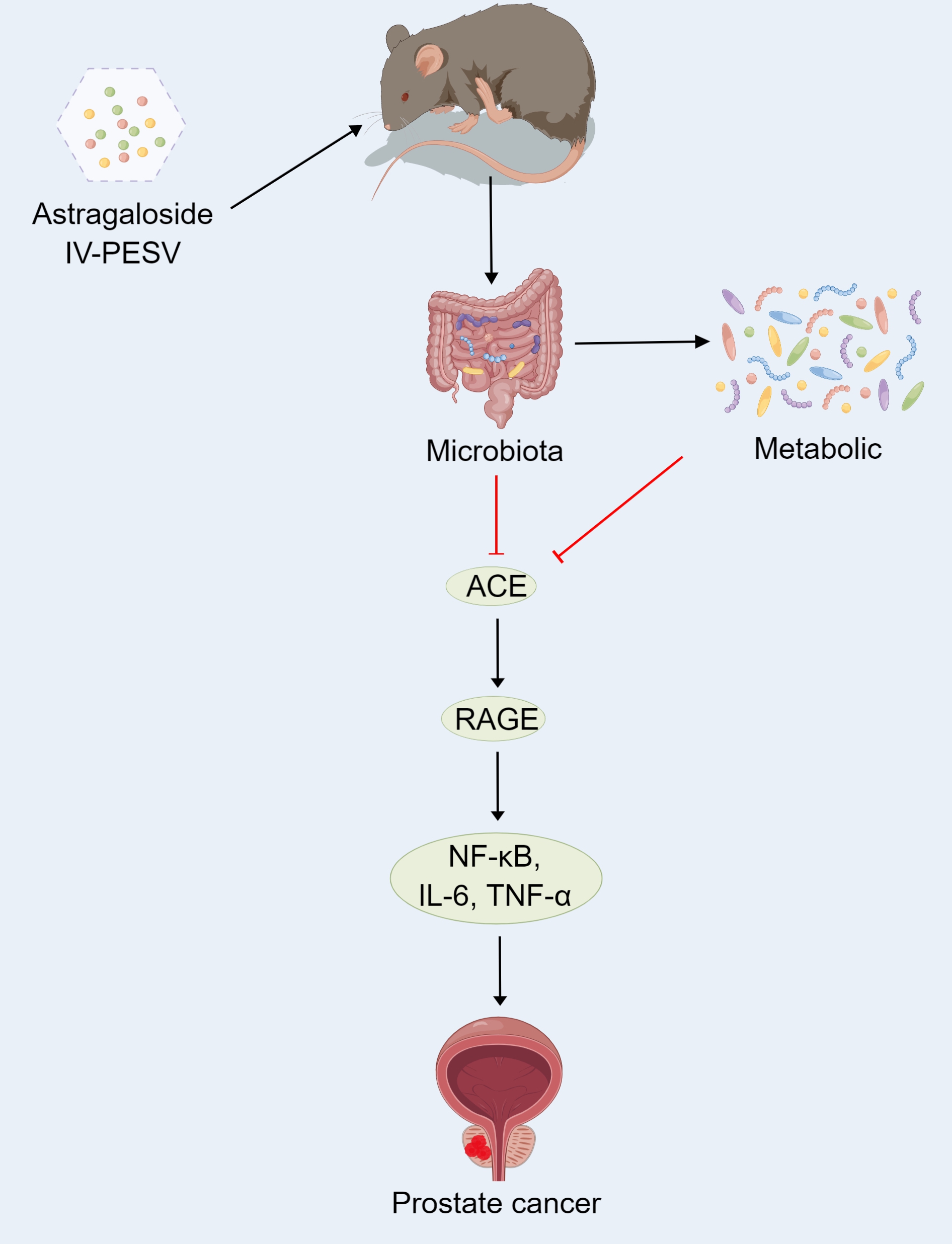

Supplement: Supplementary file 1 — Supplementary Figure S1: Astragaloside IV combined with PESV could treat PCa by intervening in gut microbiota composition and metabolite by targeting AGE-RAGE [file 12885_2024_12167_MOESM1_ESM.jpg]

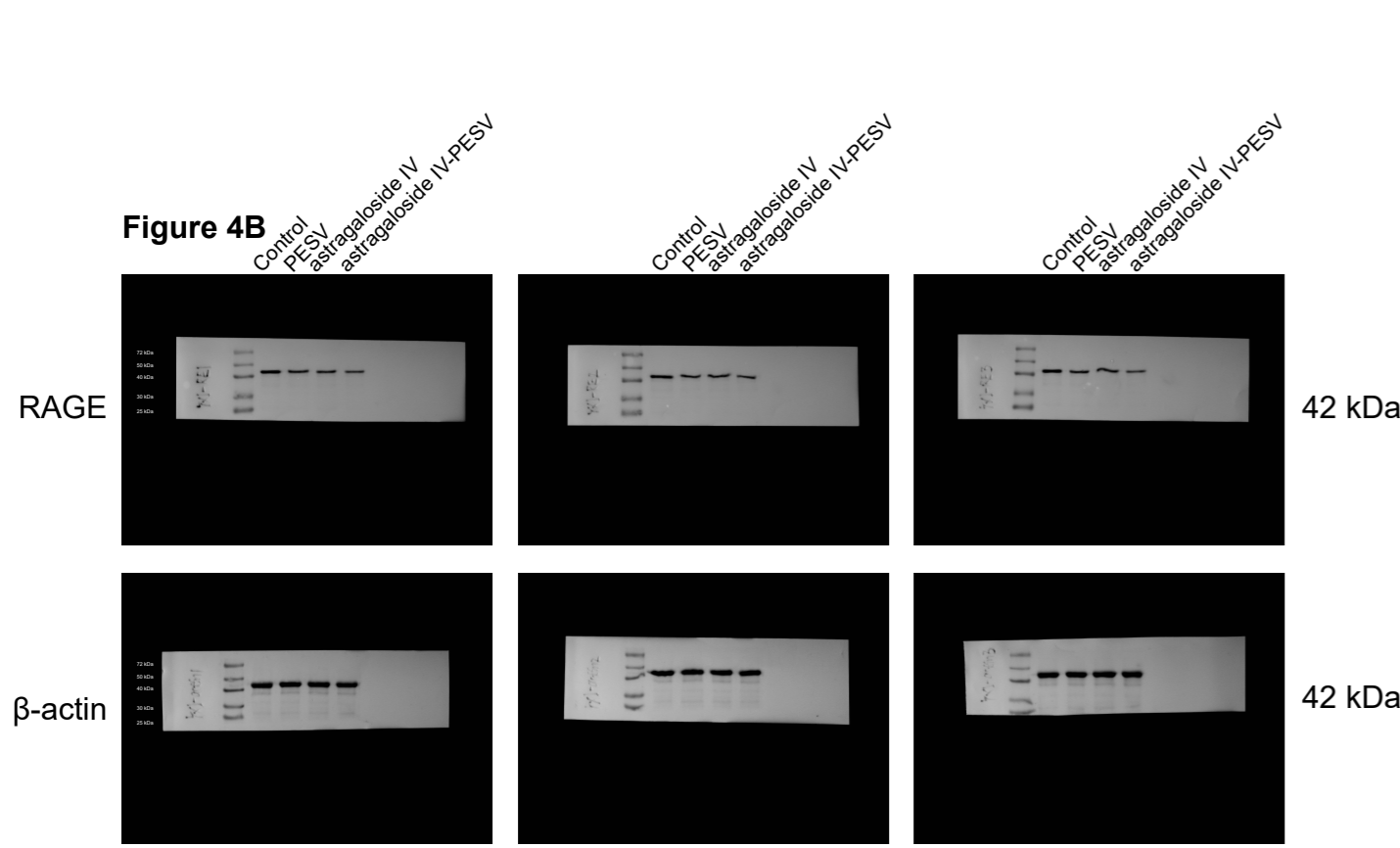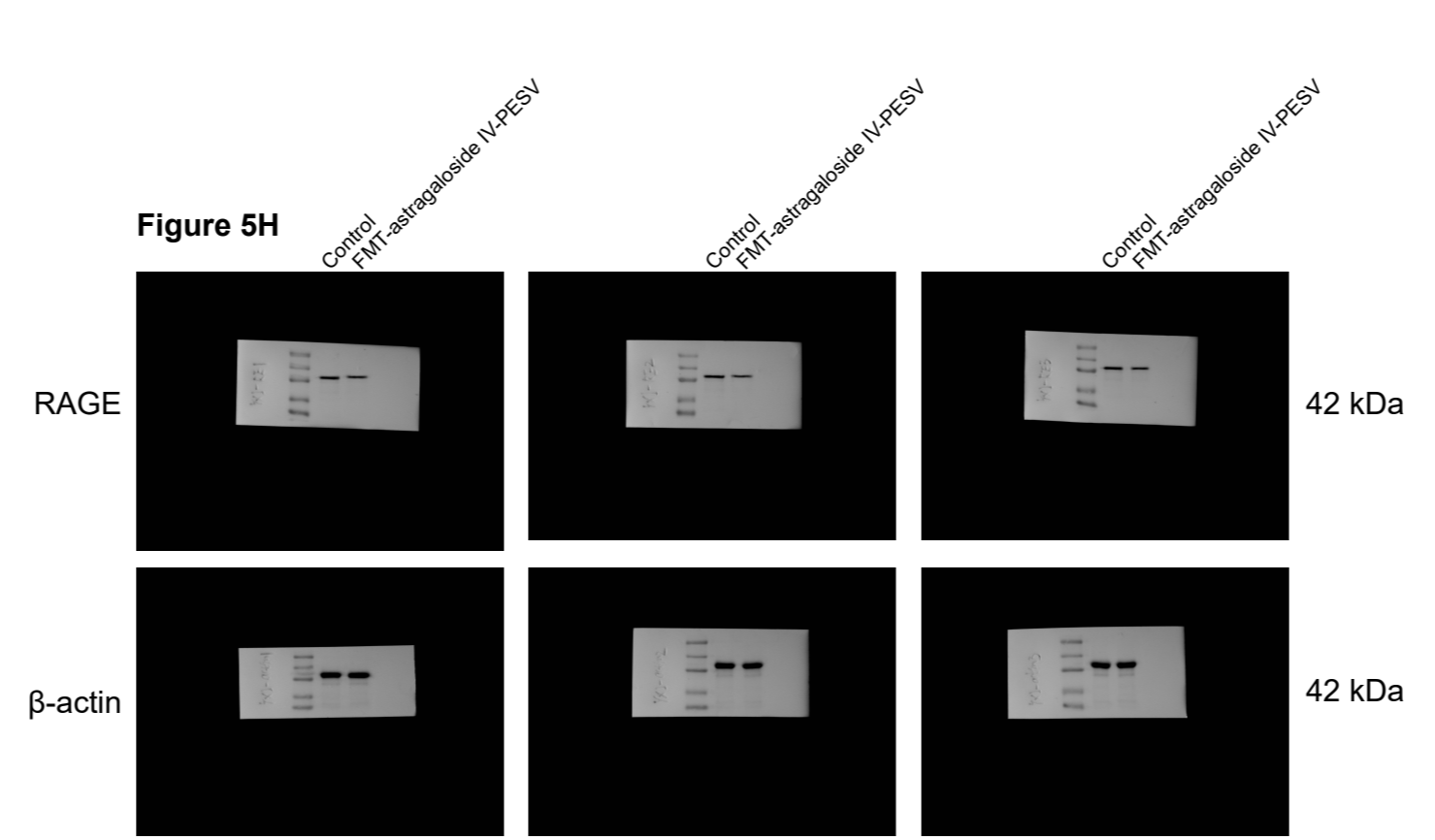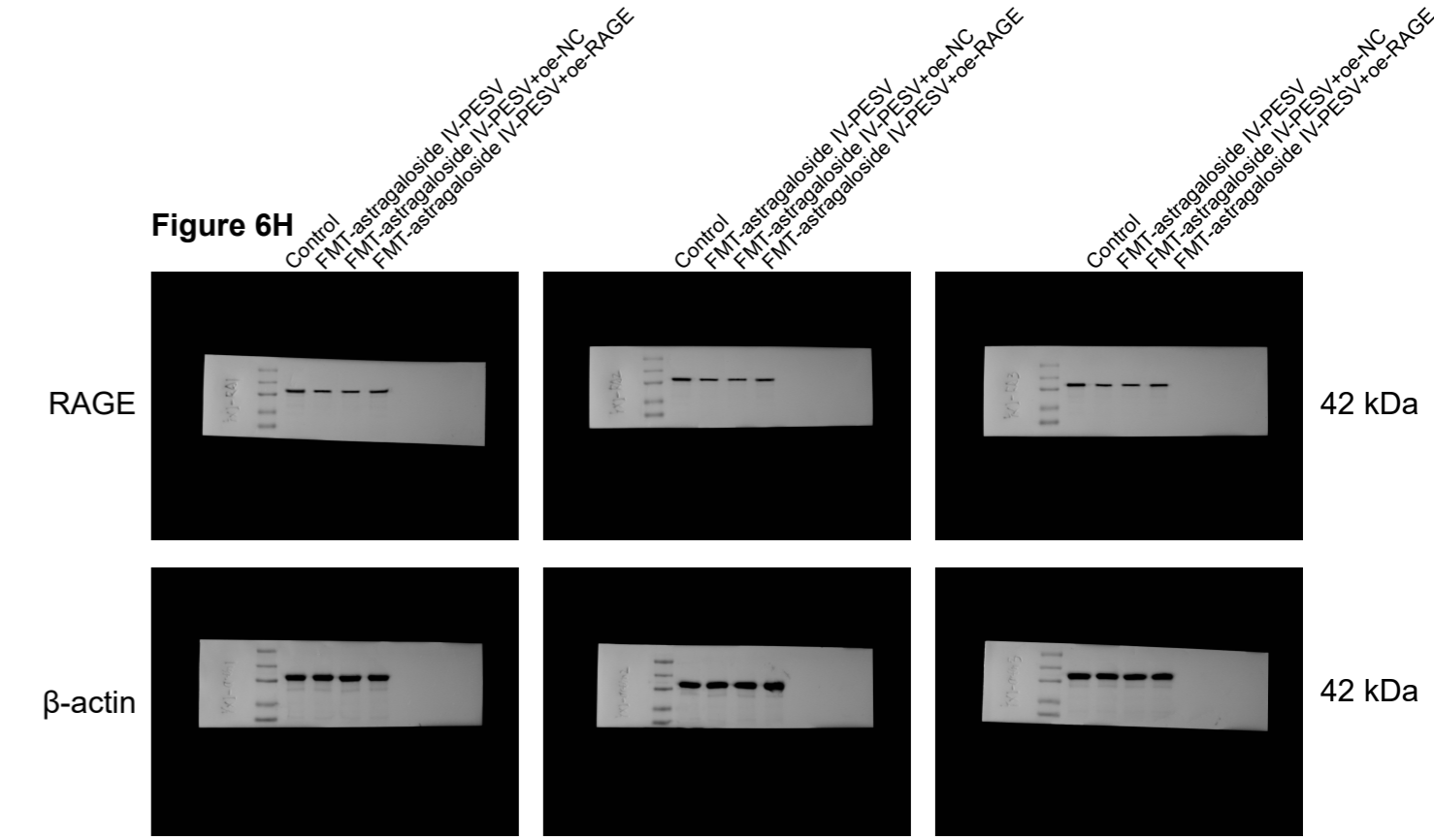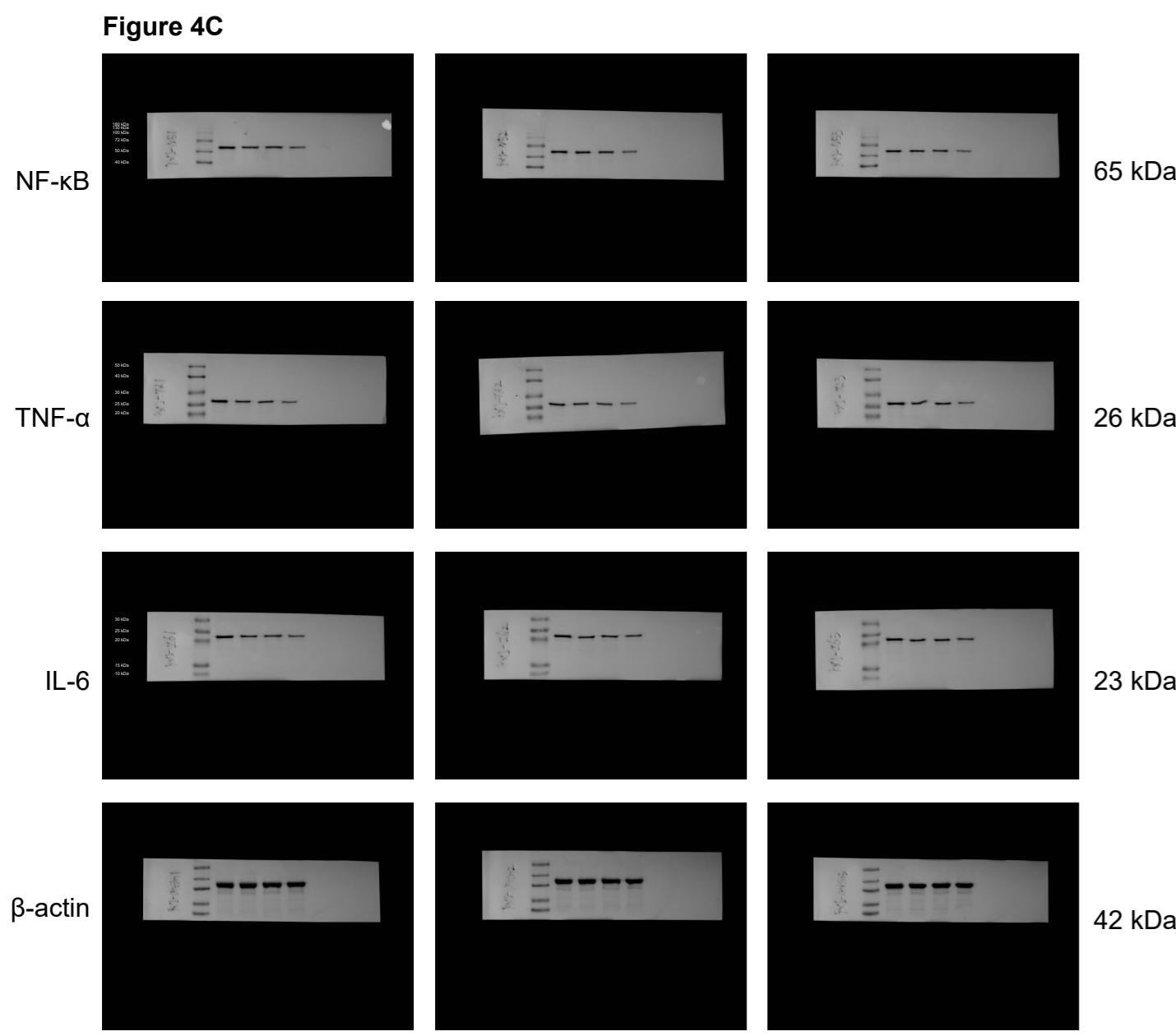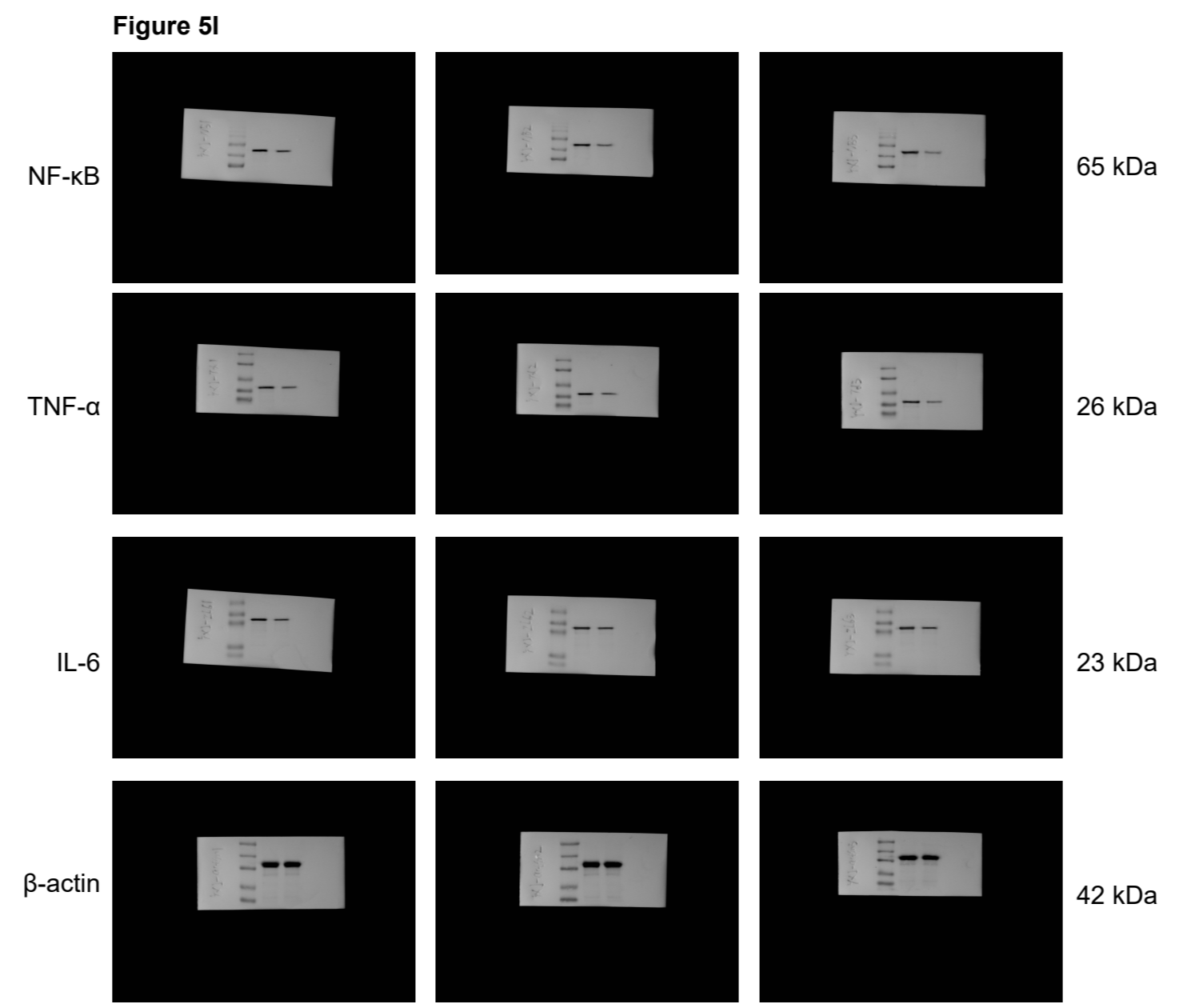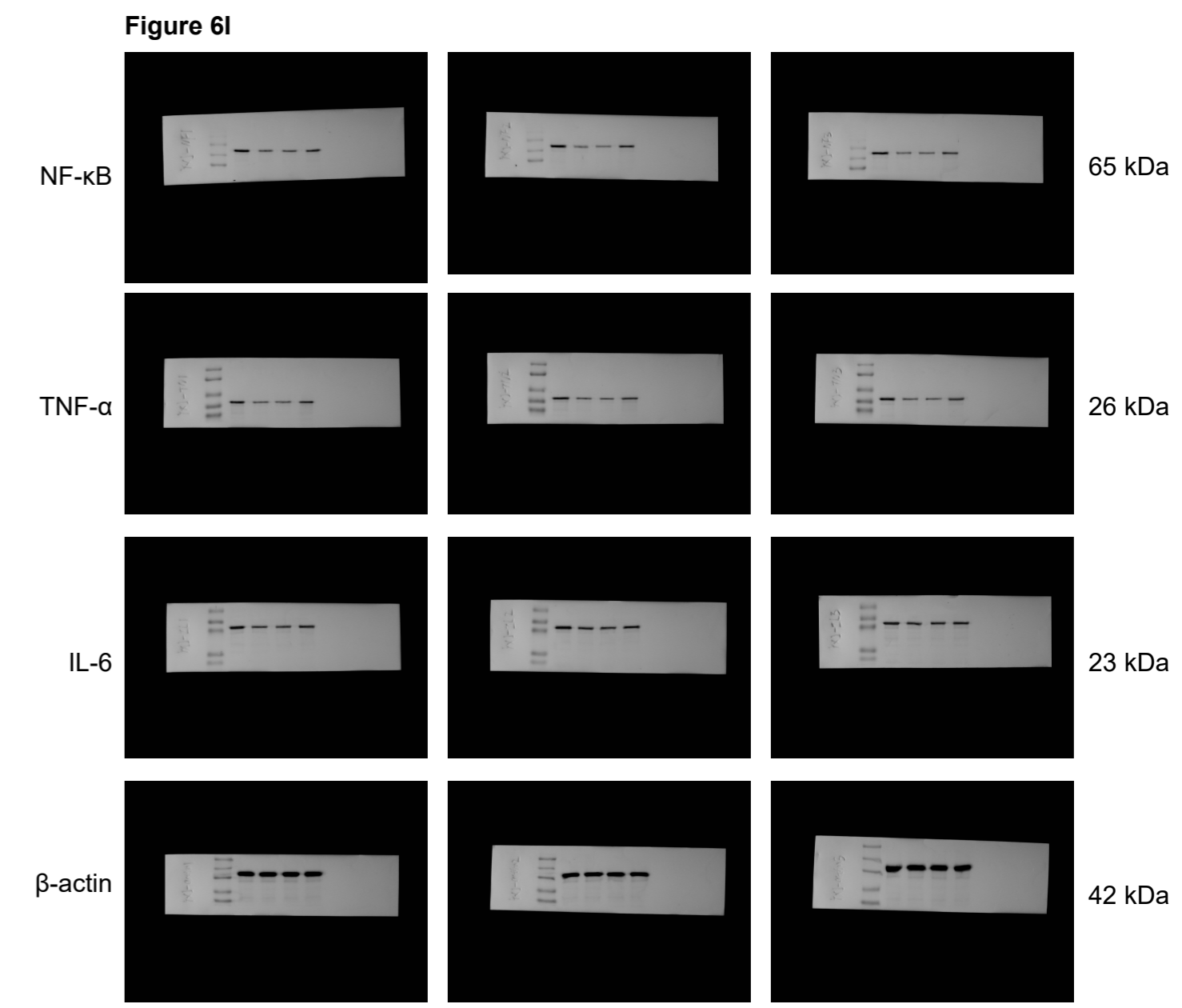

Supplement: Supplementary file 3 — Supplementary Material 3 [file 12885_2024_12167_MOESM3_ESM.pdf]
